# Analgesic and antibutyrylcholinestrasic activities of the venom prepared from the Mediterranean jellyfish Pelagia noctiluca (Forsskal, 1775)

**DOI:** 10.1186/1476-0711-11-15

**Published:** 2012-06-12

**Authors:** Yosra Ayed, Afef Dellai, Hedi Ben Mansour, Hassen Bacha, Salwa Abid

**Affiliations:** 1 Laboratory for Research on Biologically Compatible Compounds (LRSBC), Faculty of Dentistry, Monastir university, Rue Avicenne, 5019, Monastir, Tunisia; 2 Laboratory for Biotechnology and Bio Geo Resources Valorisation (LBVBGR), High Institute of Biotechnology of Sidi Thabet University of Manouba, 2020, Ariana, Tunisia; 3 University of Jendouba, Cité AlFaeiz rue Jamil Boutheina, Jendouba, 8100, Tunisia

**Keywords:** *Pelagia noctiluca*, Venom, Jellyfish, Analgesic activity, Anti-Butyrylcholinesterasic activity

## Abstract

**Background:**

Toxins derived from jellyfishes have been exploited as a model for the development of new drug promising applications to treat neurodegenerative diseases. The present work is aimed to evaluate the acute toxicity of crude venom of *Pelagia noctiluca* and then to screen the analgesic and antibutyrylcholinestrasic (anti-BuChE) activities of the crude venom and its fractions.

**Methods:**

Sephadex G75 gel was used to separate crude venom of *Pelagia noctiluca*, which led to some fractions. In addition, *in vivo* analgesic and *in vitro* plasma antibutyrylcholinestrasic activities were carried out with *Pelagia* crude venom and its fractions respectively.

**Results:**

The crude venom and its fractions displayed analgesic and anti-BuChE activities at different doses without inducing acute toxicity. Fraction 2 possesses the highest analgesic and antibutyrylcholinestrasic properties. The crude venom and fraction 1 had shown to possess less significant inhibitory activity against analgesic and antibutyrylcholinestrasic models.

**Conclusions:**

Based on this study, the crude venom of *Pelagia noctiluca* is found to be a useful tool for probing pharmacological activity. The purification and the determination of chemical structures of compounds of active fractions of the venom are under investigation.

## Introduction

Cnidarians are the largest phylum of generally toxic animals. Their toxins and venoms have not received as much scientific attention as those of many terrestrial (snakes, scorpions, spiders, etc.) and some marine animals (i.e. cone snails) [1].

The jellyfish, *Pelagia noctiluca* (*P. noctiluca*) [2] is a cnidarian of the class Scyphozoa, the order Semaestomeae, and the family Pelagiidae. It is widely distributed in different parts of the Mediterranean Sea [3-5] and in the Atlantic Ocean [6]. As a member of the phylum Cnidaria, their main characteristic is the specialized cells (cnidocytes) harbouring cnidocysts. Cnidocysts, or nematocysts, contain a complex mixture of highly active and structurally diverse toxins. The cnidocyst capsules are discharged as a response to adequate chemical and mechanical stimuli elicited by prey organisms [7].

The venom of *P. noctiluca* is of protein nature and contains peptides. It is antigenic and possesses dermonecrotic and hemolytic properties [8]; Cytolytic and neurotoxic effects have also been shown by several biological assays [9-12].

Since 1960, Scientists have studied on the biochemistry, pharmacology and toxicology of jellyfish venom and most of them have tried to extract its active components as a new natural source of drugs. There are several reports on the antimicrobial activities of the jellyfish extracts, which can afford design of new antibiotics with broad-spectrum antimicrobial activity. Ovchinnikova et al [13] have discovered a new antimicrobial peptide, termed aurelin, from a scyphoid jellyfish *Aurelia aurita*. This Anti-microbial peptide exhibited activity against Gram positive and Gram-negative bacteria [13]. Thangaraj et al [14] have studied the antimicrobial activity of the crude extract of *Stichodactyla gigantea* and *Stichodactyla mertensii*. The two sea anemone extracts exhibited significant activity against all bacterial strains. Williams et al [15] have reported that the tissue extract of *Stichodactyla haddoni* showed highly antimicrobial activity. Synthetic amino-terminal peptides from the sea anemone *Stychodactyla helianthus* have shown cytolytic activity to human erythrocytes and antibacterial activity against *Escherichia coli* and *Staphylococcus aureus*[16]. It was also reported that jellyfish venom could have promising applications in cardiovascular medicine [17]. So, it is useful to study jellyfish venom for the sake of the human health. Some experiments in rats showed that jellyfish can be used to cure arthritis, back pain and to remedy fatigue [18].

In this regard, our objective in this study was to establish a procedure for extraction of *P. noctiluca* crude venom, to evaluate the acute toxicity, then, to screen the analgesic and anti-BuChE activities of the crude venom and its fractions.

## Materials and methods

### Chemicals

Lysine acetylsalicylate (ASL) was obtained from Sanofi Winthrop Pharmaceuticals (Morrisville, PA, USA). Acetic acid (glacial) was obtained from Sigma Chemical Co. (St. Louis, MO, USA). Butyrylthiocholine iodide was purchased from Quimica Clinica Aplicada S.A. (Amposta, Spain). All other chemicals used were of analytical grade.

### Preparation of nematocysts

Specimens of *P. noctiluca* were collected from the beach of Monastir, Tunisia, in May 2010, and were identified by Professor Mohamed Nejib Daly Yahia from Faculty of Sciences of Bizerte, (Bizerte, Tunisia). The tentacles were excised manually from living specimens as soon as possible after capture and were then immediately frozen at −20°C.

The nematocysts isolation method has been previously described by Arillo et al [19]. Tentacles were submerged in distilled water for 5 h at 4°C. The ratio of organic tissue to distilled water was approximately 1:5. After a complete detachment of the epidermis the tissue was removed from the suspension containing both epidermis and undischarged nematocysts deriving from the osmotic rupture of nematocysts. The nematocysts, attached to the epidermal tissue, were separated by stirring. The nematocysts suspension was repeatedly washed in distilled water and filtered through plankton nets (100, 60 and 40 μm mesh, respectively) to remove most of the tissue debris, and then centrifuged at 4°C (ALC PK 120R, 4000g for 5min). The content, purity and integrity of nematocysts (cnidocysts) were controlled microscopically, and the concentrate nematocysts were stored at −80°C until further use [20].

### Nematocysts lysis and protein extraction

Crude venom was extracted by sonication on ice (Sonoplus, 70mHz, 30 times, 20s) of nematocysts as described by Marino et al [20]. After sonication, the suspension was centrifuged at 11,000 rpm for 5 min at 4°C. The supernatant was carefully removed, filtered and lyophilized.

### Protein determination

The protein content of the crude venom was determined according to the Bradford method (BioRad Labs, Hercules, CA) [21]. In the following, all mention of “venom and fractions concentrations” refers to protein concentration expressed in units of μg ml^-1^.

### SDS-PAGE

Protein species were observed by polyacrylamide gel electrophoresis (SDS-PAGE) as described previously [22]. Jellyfish crude venom protein (200 μg) were diluted (1:1) with sample buffer (50 mM Tris pH 6.8, 2% SDS, 20% glycerol, 2% 2-mercaptoethanol and 0.04% bromophenol blue) and were then boiled for 3 min. Running gels of 5% acrylamide and stacking gels of 12% acrylamide were used. The gels were stained with Coomassie R-250. The molecular size marker, 6–170 kDa (protein standards, Invitrogen, CA, USA), was run parallel with crude venom sample for molecular weight estimation.

### Size exclusion chromatography

About 300 mg of crude venom of *P. noctiluca* was dissolved in filtered–degassed double-distilled water. After centrifugation at 17 000 g for 15 min at 4°C, the supernatant was loaded on Sephadex G-75 gel-filtration chromatography columns (2.6 x 100 cm; Pharmacia), previously equilibrated with 200 mM ammonium acetate, pH 6.8 and eluted under the same conditions. The flow rate was 3 ml/min using a Bio-Rad 2110 fraction collector and the elution of the proteins was monitored at 280 nm by an ultraviolet detector.

### Animals

Swiss albino mice (weighing 18–25 g) of both sexes were obtained from Pasteur institute (Tunis, Tunisia). They were housed in polypropylene cages and were left for 1 week under constant conditions of temperature (22 ± 2°C) and a light/dark cycle of 12 h/12 h. Animals had free access to standard pellet diet and water *ad libitum*. Housing conditions and *in vivo* experiments were approved according to the guidelines established by the European Union on Animal Care (CFE Council (86/609)). The mice were used for the acute toxicity testing and for the analgesic investigation. Animals were divided into drug-treated ‘test’ and saline-treated ‘control’ groups of six or eight animals per group.

### Acute toxicity

For acute toxicity, mice were divided into groups of eight animals each. One group served as a control and received 0.9% NaCl alone (10 ml/kg) given intraperitoneally (i.p.), while the remaining groups were treated with increasing doses of *P. noctiluca* venom: 1, 5, 10, 20, 30, 40 and 50 mg/kg (i.p.), respectively. The mortality rate within a 48 h period was determined and the LD50 (the amount required to kill 50% of animals) was estimated according to the method described by Miller and Tainter [23]. According to the results of acute toxicity test, doses were chosen for pharmacological evaluations. After the last observation the mice were killed and the liver, lungs, heart, spleen and kidneys were withdrawn and stocked for next evaluations.

### Analgesic activity

Analgesic activity was performed according to the method of Koster et al [24] and assessed by the acetic acid abdominal constriction test (writhing test), a chemical visceral pain model. Swiss albino mice were selected 1 day prior to each test and were divided into groups of six mice each. One group served as the control and was pretreated under cutaneously with 10 ml/kg of saline. Another group was pretreated with the reference drug, acetyl salicylate of lysine (ASL), 200 mg/kg, by the same route. The remaining groups were injected intraperitoneally with 10 ml/kg of 1% acetic acid solution 30 min after the administration of the crude venom of *P. noctiluca* and its two fractions at the doses of 1 and 2 mg/kg b.w. (body weight); after acetic acid administration, the number of writhes was counted during 30 min. Antinociceptive activity was expressed as inhibition percent of the usual number of writhes observed in control animals. The percentages of inhibition were calculated according to the following formula: % inhibition = ((number of writhes)_control_ − (number of writhes)_treated group_) × 100/(number of writhes)_control_.

### In vitro Butyrylcholinesterase inhibition assay

#### Human plasma preparation

Human blood from anonymous healthy male subject (27 years) was provided by the Hospital of Monastir in Tunisia. Blood was collected in EDTA treated (1 mg/ml) glass tubes, the red blood cells were eliminated by centrifugation at 2000 g for 10 min, the plasma (supernatant) was then recuperated and diluted (1/200) with 50 mM phosphate buffer (pH = 7.4). Plasma was used immediately for studying butyrylcholinesterase activity or conserved at 2-8°C (stable for 7 days).

#### Butyrylcholinesterase inhibition assay

Butyrylcholinesterase (BuChE) inhibiting activity was measured by the spectrophotometric method previously reported by Ellman et al [25], modified by Ortega et al [26] and adapted according to our experimental conditions. Butyrylthiocholine iodide was used as substrate to assay butyrylcholinesterase activity. In order to calculate the activity of the obtained butyrylcholinesterase, the following procedure was employed: 1.5 ml of phosphate buffer 50 mM pH = 7.2, containing 0.26 mM of 5,5’-dithiobis-2- nitrobenzoic acid (DTNB), 10 μl of human plasma and 10 μl of the crude venom of *P. noctiluca* and the tested fractions (1, 10 and 100 μg/ml as final concentrations) were placed in a microcuvette, which was incubated for 15 min at 30°C. The hydrolysis of butyrylthiocholine was monitored by the formation of yellow 5-thio-2-nitrobenzoate anions resulting from the reaction of DTNB with the thiocholine released by the enzymatic hydrolysis of butyrylthiocholine. Absorbance was measured using an M350 double Beam UV–VIS spectrophotometer « Camespec » at 405 nm, and the reading was repeated during 75 s at intervals of 30 s to verify the linearity of the reaction. The enzymatic activity was calculated using the absorption coefficient 23460 and according to the relation:

(1) $$Enzymatic\;activity\;\left(UI\;\left(international\;units\right)/l\right)=23460\times \left(DO_{405nm}t_{0s}–DO_{405nm}t_{75s}\right)\text{.}$$

The percentage (%) of inhibition of BuChE activity was calculated as follows: (E – S)/E × 100. Where E is the activity of the enzyme without test compound (in our case E = 9 000 UI/l) and S is the activity of enzyme with test compound.

IC_50_ (concentrations of test compounds that inhibited the hydrolysis of substrate (butyrylthiocholine) by 50%) values were calculated from dose-inhibition curves [27]. All experiments were repeated three times.

### Statistical analysis

Data were expressed as the mean ± standard deviation of three independent experiments. The statistical analyses were performed with SPSS™ software v.10.0 (from SPSS Inc.). Data were analyzed for statistical significance using Dunnett’s test.

## Results

### SDS–PAGE of *P. noctiluca* Crude venom

**Figure 1:**
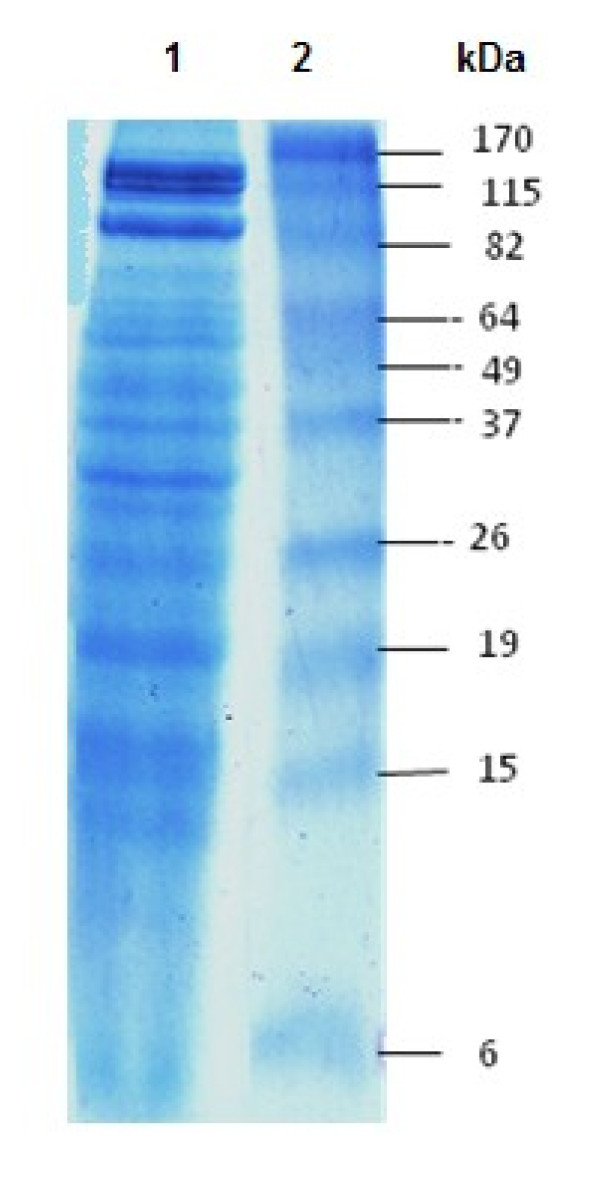
**SDS-PAGE separation of*P. noctiluca*crude venom proteins.** Electrophoresis was carried out according to Laemmli (1970) using 12% polyacrylamide gel.

Electrophoretical analysis of *P. noctiluca* crude venom revealed a number of bands of varying size (Figure 1). 15 bands appeared after staining of the SDS–PAGE gel. The molecular weights of these bands were 4; 14; 16 ; 20; 29 ; 33 ; 37 ; 45 ; 55 ; 64 ; 66 ; 70 ; 80 ; 115 and 120 kDa, respectively. The protein components of jellyfish crude venom were complex.

### Sephadex G-75 chromatography

**Figure 2:**
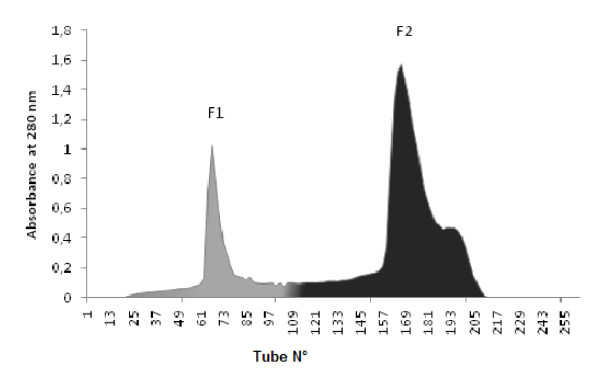
**Fractionation of** ***P. noctiluca*** **crude venom on Sephadex G-75.**

Proteins with molecular weights greater than 70 KDa are completely excluded. About 200 mg of crude venom of *P. noctiluca* were applied to a column packed with Sephadex G-75 (Pharmacia, Uppsala, Sweden) at a flow rate of 18 ml per hr gave rise to two peaks in the elution profile monitored at 280 nm (Figure 2). The two peaks were collected and named fraction 1 (F1) and fraction 2 (F2).

### Toxicity studies

Swiss albino mice were observed during 48 h and morbidity and/or mortality were recorded, for each group at the end of observation period. The LD50 of the crude venom was about 20 mg/kg b.w.

### Analgesic activity

The inhibition percentages of writhing for *P. noctiluca* crude venom and its two fractions (F1 and F2) are shown in Table 1. The reference drug acetyl salicylate of lysine (ASL) inhibited 61.88% of the number of writhing elicited by acetic acid. The analgesic effect was tested using the crude venom, F1 and F2 at the concentrations of 1 and 2 mg/kg b.w. The administration of all tested extracts induced a dose-dependent antinociceptive activity on Swiss albino mice. F2 possesses the highest analgesic properties, at 2 mg/kg b. w. (83.15%). The crude venom of *P. noctiluca* was also active; the percentage of inhibition of writhing was 77.89% at 2 mg/kg b. w. The lowest activity was observed with F1.

**Table 1:** **Analgesic activity of** ***P. noctiluca*** **crude venom, its two fractions (F1 and F2) and the reference drug (ASL)**

**Groups**	**Dose (mg/kg)**	**Number of writhes**	**Inhibition of writhing (%)**
**Control**	-	95±8.4	-
**Venom**	1	57±4.2**	40
	2	21±2.8**	77.89
**Fraction 1**	1	35± 4.2**	63.15
	2	10± 2.8**	89.47
**Fraction 2**	1	16± 1.4**	83.15
	2	4±1.4**	95.78
**Reference drug (ASL)**	200	28.33± 2.06**	70.17

Values are expressed as mean ± S.E.M. (Standard Error of the Mean) (N = 6); ASL: acetyl salicylate of lysine. Significant difference obtained with: **P < 0.01.

### *In vitro* butyrylcholinesterase inhibition effect

Results of human plasma BuChE inhibitory activity of the crude venom of *P. noctiluca* and its fractions are shown in Table 2. The crude venom and its fractions were found to inhibit the BuChE activity. The inhibition was immediate, as evidenced by the linearity of the absorbance vs. time during the 75 s assay period (r2 > 0.978). The inhibition level of BuChE activity increased significantly in presence of F2 compared to the crude venom of *P. noctiluca*. At the highest concentration (100μg/ml) of F2 and the crude venom, the inhibition was 68 % ± 4.25 and 56.44 % ± 2 respectively. The determined IC_50_ were respectively 58 and 75.5 μg/ml for F2 and the crude venom. However, F1 was the least effective as inhibitor of enzymatic activity.

**Table 2:** **Percentage of inhibitions of butyrylcholinesterase activity by:** ***P. noctiluca*** **crude venom and its two fractions (F1 and F2)**

	**Concentration (μℊ/ml)**	**Inhibition (%) against BuChe**	**IC**_**50**_** (μℊ/ml)**
Venom	1	19.51 ± 1.20*	77.5
	10	29.5 ± 1.2*	
	100	56.44 ± 2*	
F1	1	10.2 ± 2.5*	82
	10	24 ± 4.25*	
	100	54.16 ± 1.5*	
F2	1	12.35 ± 0.75*	58
	10	32.25 ± 2.35*	
	100	68 ± 4.25*	
(a) Galanthamine	1	44.5 ± 1.00**	7.9
	10	59.44 ± 2.5**	
	100	67.5 ±2.5*	

Significant difference obtained with: *P < 0.05: **P < 0.01. The reported comparisons concern: *P. noctiluca* crude venom versus control (a), Fraction 1 versus control (a) and Fraction 2 versus control (a). Every concentration is compared with its equivalent in the other extract. Significant difference obtained with: *P < 0.05: **P < 0.01.

## Discussion

A large number of marine organisms are known to posses bio-active substances that have tremendous pharmaceutical potential for the future [28]. However, considerable progress has been made to isolate and characterize the toxic components of marine cnidarians [29-32]. Several antimicrobial peptides have been isolated from Cnidaria [13-16]. To our knowledge, no antimicrobial activity of *P. noctiluca* venom has been reported.

Fortunately, we could obtain adequate *P. noctiluca* material because of the exceptional abundance of this jellyfish in the coastal water of Tunisia, in these recent years. The extraction of crude venom from nematocysts is essential before any research into crude venom toxicity can be conducted.

In an attempt to obtain crude venom from the nematocysts of *P. noctiluca*, we tried some mechanical methods including sonication, which did not destroy *P. noctiluca* nematocysts [33-35].

In the present study, Protein components of *P. noctiluca* nematocysts crude venom were determined by using SDS-PAGE on 12% polyacrylamide gel. 15 distinct clear bands were determined with molecular weight of 4; 14; 16 ; 20; 29 ; 33 ; 37 ; 45 ; 55 ; 64 ; 66 ; 70 ; 80 ; 115 and 120 kDa in *P. noctiluca* crude venom (Figure 1). It has been reported that toxic fractions of *P. noctiluca* crude venom has a molecular weight of 54, 92, 130 and 150 kDa [36].

Separation of crude crude venom of *P. noctiluca* was achieved by a size exclusion chromatography (sephadex G 75). This gel is a dextran capable of separating proteins with molecular weights between 3 and 70 KDa. The volume outside the gel matrix is known as the void volume (Vo). This is the volume required to elute a substance so large that it cannot penetrate the pores at all. Proteins with molecular weights greater than 70 KDa are completely excluded. Two peaks were collected and screened for their analgesic and BuChE inhibitory activities (Figure 2).

Great progress has been made in the last 30 years toward understanding the neural substrates of pain and identifying novel molecular targets for analgesic drug development [37]. Acetic acid induced writhing method is a sensitive procedure in detecting analgesic effect of medicinal agents [38]. *P. noctiluca* crude venom and its fractions (F1 and F2) showed significant analgesic action compared to the reference drug acetyl salicylate of lysine (ASL). The fraction 2 was found to exhibit higher analgesic activity than fraction 1 against acetic acid induced pain in mice at two dose levels (1 & 2 mg/kg b.w.), these doses were not toxic for mice.

Suganthi et al [17] reported that intraperitoneally administration of 200 mg/kg of *Crambionella stuhalmanni* and *Chrysaora quinquecirrha* extracts significantly inhibit acetic acid induced writhing in mice, the inhibition of writhing response and central nervous system depressing activity percentage were 35%, 55% and 90%, 95%.

The present results coincide with those reported by other authors who studied the analgesic property of *Conus lentiginosus* and *C. mutabilis*, which was 128 times more than that of paracetamol [39]. Shanmuganandam [40] reported the effectiveness of *Conus figulinus* venom on guinea pig skin as an infiltration anaesthetic agent. Salivary gland secretion of the gastropod *Conus sp.* is one of the most important venoms to possess analgesic property [41,42].

It is, therefore, assumed that central mechanisms may be involved in the observed phenomenon since the extract could elicit activities against pain model. Agents that exhibit these activities are believed to act primarily on the central nervous system.

*Stomolophus nomurai* stings produce distinctive psychiatric signs and symptoms [43,44]. The brain operation experiments show that this organ can be affected by the venom of *Chrysaora quinquecirrha*. The biphasic action, an early disorientation followed by recovery then eventual death may indicate two separate neuro-active principles in the venom [45].

Neurotoxins are components of venoms that are specifically directed against the nervous system. The molecular variety of these substances extends from low molecular weight alkaloids to peptides and complex proteins [46]. In Cnidaria the neurotoxins have been the subject of numerous studies [11,12,47]. Sanchez-Rodriguez et al [48] isolated a 120-kDa protein from the cubomedusa *Carybdea marsupialis* with a strong neurotoxic activity on marine crabs (*Ocypode quadrata*).

The principal role of cholinesterase (ChE) is the termination of nerve impulse transmission at the cholinergic synapses by rapid hydrolysis of acetylcholine (ACh). Inhibition of ChE serves as a strategy for the treatment of Alzheimer’s disease (AD), senile dementia, ataxia, myasthenia gravis and Parkinson’s disease [49,50].

In this study, the crude venom of *P. noctiluca* and its fractions (F1and F2) were tested to determine their ability as human BuChE inhibitors. The BuChE inhibition was determined using an adaptation of the method described by Ellman et al [25]. *P. noctiluca* crude venom, F 1 and F 2 exhibited moderate to good anti-BuChE activity. In fact, the best inhibitory activity was determined by the F 2, with an order of inhibition capacity: F2 > crude venom > F1.

In spite of the widespread occurrence of AChE in animals only a few AChE inhibitors from natural sources have been isolated. For example, polypeptide fasciculins from mamba venom [50] and an alkaloids such as physostigmine from ordeal or Calabar bean [51]. An ethanolic extract from a zoanthid crust coral *Parazoanthus axinellae* exhibited anticholinesterase activity [52].

The present study indicated that jellyfish *P. noctiluca* crude venom and its fractions contained peptides with specific cholinesterase inhibitory activities which might be responsible for some of the neurotoxic effects of the venom on animals and humans.

## Conclusions

In summary, we have shown for the first time that *P. noctiluca* crude venom and its fractions displayed analgesic and anti-BuChE activities at different doses without inducing acute toxicity. In the light of the obtained results, *P. noctiluca* crude venom can be considered as an effective agent to treat pain and neurodegenerative disorders such as AD. In future experiments, studies with purified fractions of *P. noctiluca* venom peptides could be conducted for further pharmacological, biological and toxicological characterization, such as the research of the antibiotic properties.

## Abbreviations

P. noctiluca, Pelagia noctiluca; F1, Fraction 1; F2, Fraction 2; ASL, Acetyl salicylate of lysine; BuChE, Butyrylcholinesterase; anti-BuChE, Antibutyrylcholinestrasic; DO, Optical density; AD, Alzheimer’s disease; ChE, Cholinesterase; ACh, Acetylcholine; AChE, Acetylcholinesterase..

## Competing interests

The authors declare that they have no competing interests.

## Authors’ contributions

YA carried out the studies, acquired the data, performed the data analysis, and drafted the manuscript. AD played a major role in the experimental procedures of this study. HbM carried out the statistical analysis. HB revised the manuscript and SA involved in the design and organization of the study and interpreted the results. All authors have read and approved the final manuscript.
